# The *in vitro* cytotoxic activity of ethno-pharmacological important plants of Darjeeling district of West Bengal against different human cancer cell lines

**DOI:** 10.1186/s12906-015-0543-5

**Published:** 2015-02-07

**Authors:** Bipransh K Tiwary, Sony Bihani, Anoop Kumar, Ranadhir Chakraborty, Runu Ghosh

**Affiliations:** Omics Laboratory, Department of Biotechnology, University of North Bengal, Raja Rammohunpur, Dist Darjeeling, West Bengal 734013 India; ANMOL Laboratory, Department of Biotechnology, University of North Bengal, Raja Rammohunpur, Dist Darjeeling, West Bengal 734013 India

**Keywords:** Darjeeling, Medicinal plants, Anticancer, Cytotoxicity, Phytochemicals

## Abstract

**Background:**

Plant derived components have attracted particular attention as an alternative source to battle several diseases including cancer. The variation in the climate, the geographical location and the rich ethnomedicinal traditions has made the Darjeeling Himalayas an abode of invaluable repository of traditional medicinal plants. In this study, we explored the *in vitro* anticancer properties of traditionally used medicinal plants from the Darjeeling hills against different human cancer cell lines.

**Methods:**

The ethanolic leaf extracts of 30 medicinal plants were tested for their cytotoxicity against human breast adenocarcinoma cell line (MCF 7), human hepatocarcinoma cell line (HepG_2_) and human cervix adenocarcinoma cell line (HeLa). The cytotoxicity was evaluated by performing MTT assay, trypan blue exclusion assay and morphological assessment under phase contrast inverted microscope. For the extracts which tested positive, IC_50_ (the concentration that inhibited cell growth by 50%) was calculated. The extract(s) were further subjected to Thin Layer Chromatography (TLC) to determine their phytochemical profile.

**Results:**

Out of the 30 plant extracts tested, five plants, *Artemisia indica*, *Eupatorium odoratum*, *Eupatorium adenophorum*, *Maesa macrophylla* and *Phlogacanthus thyrsiformis* showed a > 50% growth inhibition of cancer cell lines at a concentration of 50 μg/ml. The sensitivity to different extracts varied according to the cell type under investigation. Of these plants, *Maesa macrophylla*, exhibited the most potent cytotoxicity against HeLa and MCF7 cell with IC_50_ values of 9.55 μg/ml and 16.19 μg/ml respectively. Phytochemical analysis revealed the presence of coumarins, flavonoids, tannins, saponins, steroids and terpenes.

**Conclusions:**

This is perhaps the first report of screening of traditional medicinal plants from Darjeeling district in West Bengal, India, for their cytotoxic activity against three human cancerous cell lines MCF7, HeLa and HepG_2_. The extracts of *Maesa macrophylla* significantly inhibited the growth of HeLa and MCF7 cancerous cell lines and constituted of multiple known biologically active compounds. The present study may provide the landmark for further exploration of *M. macrophylla* for its potent anticancer constituents.

**Electronic supplementary material:**

The online version of this article (doi:10.1186/s12906-015-0543-5) contains supplementary material, which is available to authorized users.

## Background

Cancer stands second, after cardiovascular disorders, in the list of diseases responsible for maximum deaths in the world [1]. In 2008, the International Agency for Research on Cancer assessed that India represented about 8% of cancer deaths globally and about 6% of all deaths in India were due to cancer [2]. Recent studies have shown that the number of cancer deaths is increasing every year and the number is expected to increase further in future [3,4]. Despite the advancement in the cancer therapies which include chemotherapy and radiation therapy, the mortality rate associated with cancer has remained high. Thus, the present scenario and the toxic side effects associated with the available treatments calls for alternative methods, with higher efficacy and lesser noxious side effects, to deal with this disease. Plants have been looked upon as the best substitute and several have been evaluated in an effort to discover novel, potential anticancer compounds with no toxic effects [5,6]. In fact, several studies have elucidated the potential of a number of naturally derived phytochemicals as therapeutic agents against cancer. The first of these to advance into clinical use for the treatment of cancer were alkaloids, vinblastine and vincristine, isolated from the Madagascar periwinkle, *Catharanthus roseus* L. [7]. Others in this list includes Combretastatins obtained from *Combretum caffrum* [8], paclitaxel (Taxol), isolated from the Pacific yew, *Taxus brevifolia* [9] and homoharringtonine isolated from *Cephalotaxus harringtonia* [10]. In addition, numerous medicinal plants having anticancer properties have been identified [11].

Darjeeling district is situated in the northernmost part of the state of West Bengal in India that lies between 28°31′-27°13′ N latitude and 87°59′ - 88°53′ E longitude in the Eastern Himalayan region of India. The variation in altitude between hills and plains of Darjeeling that ranges between 150 m to 3636 m, has led to diverse climatic conditions that supports one of the richest biodiversity in the world. Thus, this region houses a wealth of varied medicinal plants [12]. The ethnic communities of Darjeeling hills, major being Lepcha, Bhutia and Nepalese, still depend on plant-derived resources for primary health care needs. The socio-economic status has persuaded the development of affluent ethnomedicinal traditions in this region [13]. The diversity of the medicinal plants in the Darjeeling district has been well documented and studies have been done to evaluate antibacterial activity of some of these plants [14-16]. To the best of our knowledge, the ability of these traditional medicinal plants from Darjeeling for their cytotoxic activity against cancer cell lines has not been investigated. In this study, conceivably for the first time, an attempt was made to evaluate the *in vitro* cytotoxic activity of ethanolic extracts of 30 ethno-pharmacological important plants against three different human cancer cell lines and characterize the phytochemical constituents with the view of isolating compounds from these plants which may contribute to drug development against cancer.

## Methods

### Plant material and preparation of extracts

Fresh leaves of plants as listed in Table 1 were collected from Darjeeling district of West Bengal, India. The specimens were identified and the voucher specimens were assigned specific reference number (Table 1).The leaves were washed thoroughly, air-dried and crushed. The crushed samples were extracted with 95% ethanol (1 g in 10 ml ethanol) for 72 h at 37°C with occasional shaking. After 72 h, the extract was filtered through a Whatman no. 1 filter paper and the filtrate was evaporated to dryness using a rotary evaporator (RV 10 Digital, IKA, Germany). The dried samples were stored at −20°C until use.

**Table 1:** List of plants screened for cytotoxicity with their ethnomedicinal uses in Darjeeling district*

**S. no.**	**Botanical name**	**Voucher number**	**Family**	**Local name**	**Type**	**Part used**	**Local medicinal uses**
1	*Acmella calva* (DC.) Jansen	*DJ0092*	*Asteraceae*	Kalijhar	Herb	Flower, inflorescenc	Toothache, decay, mouth sore
2	*Aconogonon molle* (D. Don) Hara	*DJ0099*	*Polygonaceae*	Thotney	Shrub	Young shoot	Astringent
3	*Acorus calamus* L.	*DJ0049*	*Araceae*	Bojho	Herb	Root/Rhizome	Vermifuge, fever antispasmodic, Insect repellent Paste prepared from dried or fresh rhizomes is applied on forehead during fever. Powder made fromdried rhizomes is administered orally in case of fever, bronchitis
4	*Artemisia indica* Willd.	*DJ0093*	*Asteraceae*	Titey pati	Herb	Leaves & Young Shoot	Use in Skin diseases, asthma, anthelminthic, stomachic, purgative, antispasmodic and amoebic dysentery
5	*Astilbe rivularis* Buch.-Ham. ex D. Don	*DJ0077*	*Saxifragaceae*	Buriokahti	Herb	Leaves/roots/Rhizome	Diarrhea, dysentery, blood purifier Root’s juice or pieces are taken orally during diarrhoea or dysentery
6	*Bauhinia vahlii* Wight & Arnott	*DJ0091*	*Caesalpiniaceae*	Verla	Climber	Seeds bark leaves	Seeds used as tonic, aphrodisiac, leaves demulcent, bark is useful in skin disease, diarrhea
7	*Bergenia ciliata* (Haw.) Sternb.	*DJ0076*	*Saxifragaceae*	Pakhanbed	Herb	Root & rhizome	Tonic, fever, boils, astringent
8	*Callicarpa arborea* Roxb.	*DJ0090*	*Lamiaceae*	Guahelo	Shrub	Bark & root	The bark juice is given to treat fever. The root is chewed in cases of boils on the gums
9	*Cedrella toona*	*DJ0057*	*Meliaceae*	Toonee	Tree	Bark, fruit, leaf, flower	It is useful in chronic dysentery, ulcer, leprosy, cures fever, headache, blood complaints, cardiotonic, aphrodisiac, anthelmentic; good for scabis and expectorant
10	*Cinchona succirubra* Pav. ex Klotzsch	*DJ0096*	*Rubiaceae*	Kulain	Shrub	Trunk & Stem bark	Malaria fever, neuralgia, sciatica
11	*Costus speciosus*	*DJ0102*	*Costaceae*	Betlaure	Herb	Root	Useful in fever, bronchitis, anemia, rheumatism and diabetic
12	*Dichroa febrifuga* Lour.	*DJ0073*	*Hydrangeaceae*	Basak	Shrub	Roots& Leaves	Fever, malaria
13	*Drymaria cordata* (L.) Willd. ex Schult	*DJ0100*	*Caryophyllaceae*	Abhijal	Herb	Whole plant	Above ground parts-steamed and smelled during sinus trouble. Plant paste for fever, cold and cough also used for dog bites, headache
14	*Equisetum debile* Roxb.	*DJ0071*	*Equisetaceae*	Kurkure Jhar	Herb	Aerial part	Clotting agent used in wound, nose bleeding & bleeding of urinary tract
15	*Eupatorium adenophorum Spreng./ 6084*	*DJ0054*	*Asteraceae*	Kalo banmara	Herb	Leaf	Uses in external cut and wound
16	*Eupatorium odoratum* L.	*DJ0089*	*Asteraceae*	Kalijhar	Herb	Aerial part	Clotting agent used in wound, nose bleeding & bleeding of urinary tract
17	*Fagopyrum dibotrys* (D. Don) Hara	*DJ0097*	*Polygonaceae*	Ban phapar	Herb	Fruit & Grains	Diet in colic, used in lungs infection and pulmonary abscess
18	*Hypericum uralum* Buch.-Ham. ex D.Don	*DJ0072*	*Hypericaceae*	Urilo	Herb	Shoot, flower & seeds	Uses in wound and bruise. Also used as Stimulant
19	*Maesa macrophyla*	*DJ0056*	*Myrsinaceae*	Boghati		Bark, fruit, leaf	Uses in tonsillitis, malarila fever, scabis, diphtheria
20	*Mesua ferrea*	*DJ0091*	*Nageeswari*	*Nageeswari*	Shrub	Bark	Uses various skin diseases (mostly poxes)& in menstrual disorder
21	*Oscbekia nepalensis*	*DJ0083*	*Melastomataceae*	Angeri	Shrub	Young leaf or tender shoot	Pneumonia, fever, common cold
22	*Phlogacanthus thyrsiformis* (Hardw.) Mabb.	*DJ0095*	*Acanthaceae*	Chuwa	Shrub	Leaf bark & inflorescence	Liver cirrhosis, body ache, piles, dysentery
23	*Pteris biaurita* L.	*DJ0067*	*Pteridaceae*	Thadey unew	Herb	Stem, leaf stalk	Bleeding and infection and dysentery
24	*Rumex nepalensis* Spreng.	*DJ0081*	*Polygonaceae*	Halhaley	Herb	Root	Root dried or fresh extract used orally in hepatitis, loss of hair, also plant used as dyes
25	*Selaginella monospora*	*DJ0059*	*Selaginellaceae*		Shrub	Leaves	Prevents cough, bleeding piles, gravel aminorrhoea
26	*Smilax zeylanica* L.	*DJ0087*	*Smilacaceae*	Kukur	Shrub	Thorny climber	Used in Urinary complaints and dysentery Roots are taken as tonic
27	*Solanum torvum* Sw.	*DJ0066*	*Solanaceae*	Jungali bihi	Herb	Leaves	Toothache and jaundice
28	*Stephania glabra* (Roxb.) Miers	*DJ0086*	*Menispermaceae*	Taubarkey	Climber	Root bulb	Powder used in diabetes, tuberculosis, asthma, fever
29	*Tetradium fraxinifolium* (Hook.f.) T.G. Hartley	*DJ0082*	*Rutaceae*	Khanakpa	Tree	Fruits, leaves	Gastritis, hepatic disorder, dysentery, Indigestion, skin disease
30	*Thysanolaena latifolia* (Roxb. ex Horn.) Honda	*DJ0065*	*Poaceae*	Amliso	Shrub	Young shoots & fresh root	Tonsillitis, boils, abortion, mouth wash skin diseases

*References [21-23,35].

### Human cell lines

The human breast adenocarcinoma cell line (MCF 7), human hepatocarcinoma cell line (HepG_2_) and human cervix adenocarcinoma cell line (HeLa) was obtained from National Centre for Cell Science, Pune, India. All cell lines were cultured in DMEM (Dulbecco’s Modified Eagle Medium) supplemented with 10% fetal bovine serum (FBS), 100 units/ml penicillin, 100 mg/ml streptomycin, 0.14% sodium bicarbonate and 0.1 mM sodium pyruvate. The cell lines were maintained in CO_2_ incubator (N-Biotech) at 37°C in a 5% CO_2_ atmosphere with 95% humidity.

### In vitro cytotoxic activity

Stock solutions at a concentration of 10 mg/ml were prepared by reconstituting the dried alcoholic extracts in dimethyl sulfoxide (DMSO, Hi-Media). The cytotoxic effect of each of the plant leaf extract was evaluated by tetrazolium- dye, MTT, assay [17,18] with slight modifications. Briefly, the cancerous cells (MCF 7, HepG_2_ and HeLa) were seeded in 96-well plates at a density of 5×10^3^ cells/well in 200 μl culture medium. Following 24 h incubation and attachment, the cells were treated with different concentrations of extracts and similar concentration of diluents (DMSO) for further 24 h. After treatment, media was replaced with MTT solution (10 μl of 5 mg/ml per well) prepared in PBS and incubated for 3 h at 37°C in a humidified incubator with 5% CO_2_. The yellow MTT dye was reduced by succinate dehydrogenase in the mitochondria of viable cells to purple formazan crystals. To solubilize the formazan, 50 μl of isopropanol was added to each well. The plates were gently shaken for 1 min and absorbance was measured at 600 nm, with reference 490 nm, by microtiter plate reader (MIOS Junior, Merck). The percentage of cytotoxicity was calculated as (Y-X)/Y x 100, where Y is the mean optical density of control (DMSO treated cells) and X is the mean optical density of treated cells with plant extracts.

### Morphological assessment of cancerous cells

Cells were seeded in 35 mm polyvinyl coated cell culture plates and allowed to attach at 37°C for 24 h in CO_2_ incubator. The following day, cells were treated with either 30 μg/ml plant leave extract or DMSO alone, serving as control, and incubated again at the same conditions. The morphological changes of cancerous cells under treated and control conditions were compared by monitoring with phase contrast inverted microscope (Olympus, CK40-SLP) at 200X magnification. The images were photographed at 8, 16, and 24 h of incubation.

### Trypan blue exclusion assay

After morphological assessment, the cell viability was simultaneously assessed by Trypan blue dye exclusion assay [19]. For this, the cells were trypsinized with 0.25% trypsin-EDTA solution, resuspended in phosphate buffer saline (PBS) and stained with 0.4% Trypan blue dye solution (v/v in PBS). Within two minutes, the cells were loaded in a Neubauer chamber and the number of viable and non-viable cells per 1 x 1 mm square was counted under phase contrast microscope. The dead cells, because of losing the semi permeability of membrane, retained the blue dye and hence are coloured whereas viable or live cells remained unstained. The cells/ml was calculated as average cell count x dilution factor x 10^4^ cells/ml. The % cell viability was determined as [(no. of viable cells/ total no. of viable + non-viable cells) x 100. The percentage of growth inhibition was represented as {cell viability (control) – cell viability (with extract)}.

### Thin layer chromatography (TLC) and phytochemical analysis

Phytochemical screening by means of TLC was carried out for selected plants following the method of Wagner and Bladt [20]. For this, the dried extracts were reconstituted in ethanol to a concentration of 10 mg/ml. 20 μl of the extracts were spotted, in triplicate, on aluminium-backed TLC plates (Merck, silica gel 60 F254). The chemical constituents were separated using any one of the three different eluent systems. For polar/neutral eluting system, solvents used were ethyl acetate/methanol/water (40:5.4:5); for intermediate polarity/acidic elution, solvents ethyl acetate/formic acid/glacial acetic acid/water (10:1.1:1.1:0.5) were used; and for non-polar/basic elution, hexane/ethyl acetate (3:1) solvents were used. The TLC plates were dried under a stream of cold air until there was no solvent smell remaining to ensure complete removal of the eluting solvents. The plates were examined under UV light (365 nm) to detect coumarins that appear as blue, violet or yellow fluorescent spots. The specific groups present in the extracts were identified using specific developers. Vanillin-sulphuric acid spray reagent (0.1 g vanillin:28 ml methanol:1 ml sulphuric acid) were then sprayed on the dried plates and heated at 110°C for colour development for detecting the presence of monoterpene alcohol, bitter principle and saponin. Sprinkling the plates with 5% ethanolic solution of aluminum Chloride (AlCl_3_) resulting in the appearance of yellow or greenish fluorescent spot under UV light at 365 nm indicated the presence of flavonoids. A brown or yellowish coloration with Lieberman-Burchard’s reagent reveals the presence of triterpenes and steroids. A 10% vanillin ethanol solution was used for detecting saponins, the presence of which results in blue, violet and sometimes yellow spots. Using a 2% ferric chloride solution, development of chestnut, violet, green and blue colour reveals the presence of polyphenols, whereas a bluish or greenish black colour would indicate a positive test for tannins.

### Statistical analysis

Results were presented as Mean ± SD of triplicates from three independent experiments. To calculate the concentration required to produce 50 % reduction in cell viability (IC_50_) regression analysis was performed using Microsoft Excel 2010. Data were analyzed and compared by one-way analysis of variances (ANOVA) by using the software SPSS 15.0 for windows (SPSS Inc. Chicago, IL, USA) and differences with p < 0.05 were considered significant.

## Results and discussion

The medicinal plants are the most sought after substitutes as the source of antimicrobial and anticancer chemotherapeutic agents. The use of traditional medicinal plants for varied ailments by different ethnic groups of Darjeeling district has been aptly recognized and it has been suggested that cures for diseases like cancer; AIDS, etc. may lay hidden in this wealth of folklore medicinal plants [13,21,22]. We collected 30 plants that have been reported to be used as cure for a wide range of diseases as has been summarized in Table 1, with the aim to uncover their cytotoxic properties. Recent reports have shown that cancer frequency in West Bengal has drastically increased and the most frequently affected organs included breast, cervix, lung, liver and pancreas [23]. Thus, for screening studies, we used three human cancerous cell lines, MCF7 (breast adenocarcinoma), HepG_2_ (hepatocarcinoma) and HeLa (cervix adenocarcinoma), as high rates of these specific cancers were noted in West Bengal. Several studies on screening various folklore medicinal plants to identify their cytotoxic activity against human carcinoma cell lines have been reported [24–27].

Ethanolic leaf extract of each of the listed plants were screened for their cytotoxicity against the three cancer cell lines at a single dose of 50 μg/ml. Our aim was to consider only those plants which exhibited a potent cytotoxic activity (~50%) and to meet this need we assessed the growth inhibitory activity of 50 μg/ml of each extract at an incubation period of 24 h. Preliminary screening results showed that the extent of inhibition varied between the types of cell lines and also the types of extract used. Out of the 30 extracts tested, 9 had almost no effect (<10 %) on the growth of any of the three cell lines (Table 2). An inhibition of < 50 % and >11 % (moderate cytotoxic activity) of 11 plant extracts against HeLa cells, 6 plant extracts against HepG_2_ cells and 2 plant extracts against MCF7 cells was observed. From the total of 30 plant extracts, only five showed > 50 % cytotoxic activity against the different cancerous cell lines at a concentration of 50 μg/ml after 24 h incubation. The possibility that the other plant extracts may also show greater inhibitory activity at either higher concentration or prolonged incubation cannot be ruled out. Nevertheless, we set stringent conditions to identify extracts exhibiting highly potential and effective cytotoxicity. In a nutshell, results of our study implicated that the human cervix adenocarcinoma cell line (HeLa) was sensitive while the other two cell lines, human breast adenocarcinoma (MCF7) and human hepatocarcinoma cell line (HepG_2_) were comparatively resistant to cytotoxicity of the tested extracts. Similarly, while screening plants used in Thai folklore medicine for cytotoxic activity, Mahavorasirikul *et al*. observed that sensitivity towards the tested extracts was dependent on the type of cancer cell line used and HepG_2_ appeared to be the most resistant cell line [27]. In addition, it may also be hypothesized that the variation may be due to the tissue specificity of different components present in the extract [28].

**Table 2:** **Percentage inhibition in the growth of HeLa, HepG2 and MCF7 cancer cell lines in the presence of 50 μg/ml of ethanolic extracts of leaves of the plants**

**S. no.**	**Plant name**	**HepG2**	**HeLa**	**MCF7**
1	*Acmella calva*	0	2.95 ± 0.81	0
2	*Aconogonon molle*	0	4.91 ± 1.25	0
3	*Acorus calamus* L.	0.23 ± .04	0	0
4	*Artemisia indica*	44.61 ± 1.9	59.34 ± 0.28	58.869 ± 0.25
5	*Astilbe rivularis*	49.34 ± 1.5	0	0
6	*Bauhinia vahlii*	0	35.08 ± 2.68	0
7	*Bergenia ciliata*	27.14 ± 4.8	6.23 ± 2.3	0
8	*Callicarpa arborea*.	0	23.93 ± 2.96	0
9	*Cedrella toone*	0	28.52 ± 3.3	0
10	*Cinchona succirubra*	0	0	0
11	*Cortus speciosus*	0	47.87 ± 1.2	1.32 ± 0.6
12	*Dichroa febrifuga*	0	0	19.55 ± 0.67
13	*Drymaria cordata* L.	0	0	0
14	*Equisetum debile*	0	15.73 ± 2.3	0
15	*Eupatorium adenophorum*	0	60.98 ± 1.7	0
16	*Eupatorium odoratum* L.	53.11 ± 4.87	78.03 ± 1.63	23.82 ± 5.35
17	*Fagopyrum dibotrys*	0	35.74 ± 0.98	0
18	*Hypericum uralum*	0	0	0
19	*Maesa macrophylla*	24.31 ± 3.1	80.98 ± 1.2	74.33 ± 0.16
20	*Mesua ferrea*	3.54 ± 0.9	0	0
21	*Oscbekia nepalensis*	0	1.63 ± 0.84	0
22	*Phlogacanthus thyrsiformis*	0	58.03 ± 4.2	52.34 ± 1.8
23	*Pteris biaurita* L.	34.23 ± 3.0	46.56 ± 0.35	0
24	*Rumex nepalensis*	0	41.97 ± 1.34	0
25	*Seleginella monospora*	0	0	0
26	*Smilax zeylanica* L.	0	33.44 ± 0.35	0
27	*Solanum torvum*	0	11.14 ± 1.2	0
28	*Stephania glabra*	0	14.75 ± 2.5	0
29	*Tetradium fraxinifolium*	0	0	0
30	*Thysanolaena latifolia*	29.50 ± 3.9	0	0

Data are presented as mean ± SD from three independent experiments, each run in triplicate. The % inhibition was calculated as percent difference between growth in DMSO (control) and growth in the presence of extracts after 24 h of incubation (treated).

**Figure 1:**
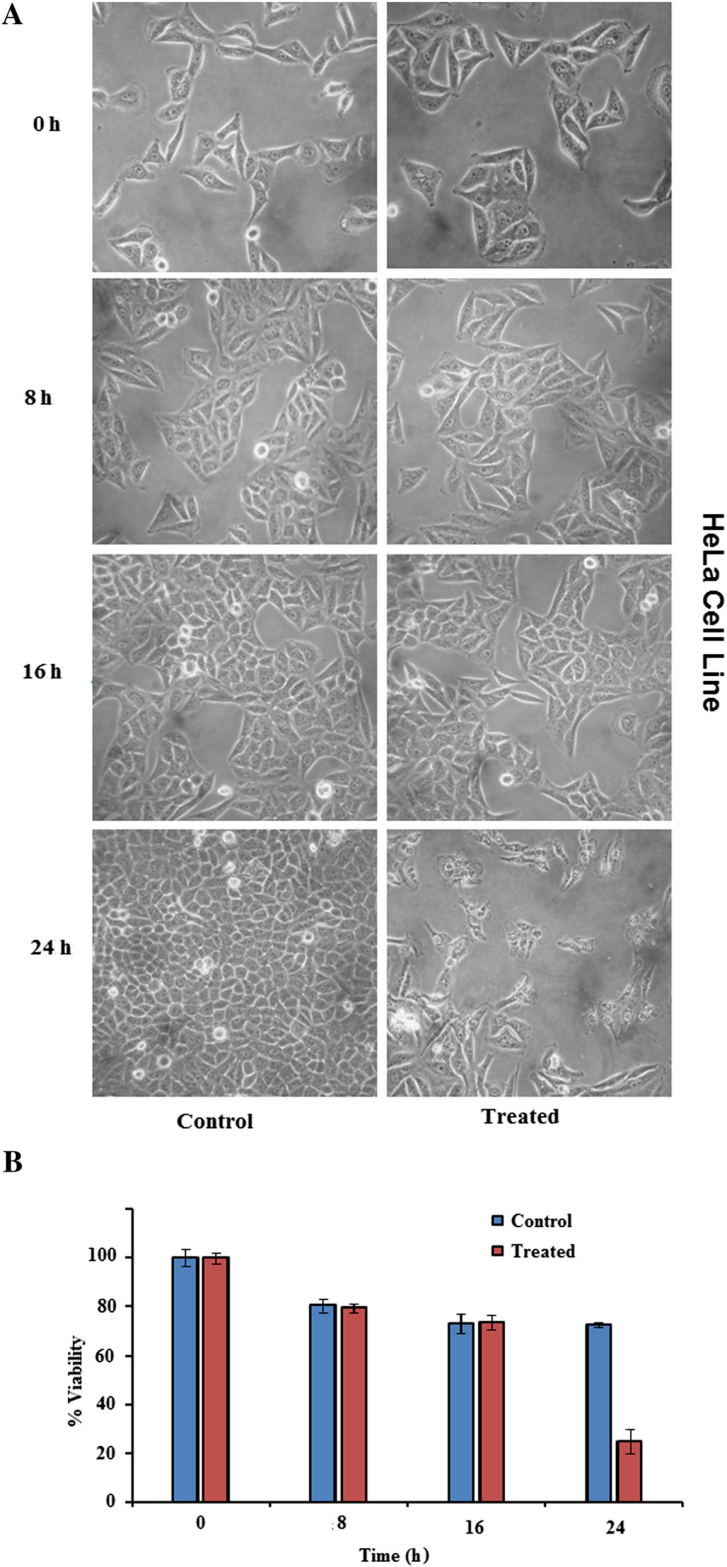
**Time dependent effect of 30 μg/ml of** ***M. macrophylla*** **leaf extract on the proliferation of HeLa, human cervix adenocarcinoma, cell line.** The cells were incubated either with DMSO (control) or the extract (treated) and **(A)** observed under phase contrast microscope; and **(B)** % inhibition in growth was quantified based on Trypan blue dye exclusion assay; at different time points.

**Figure 2:**
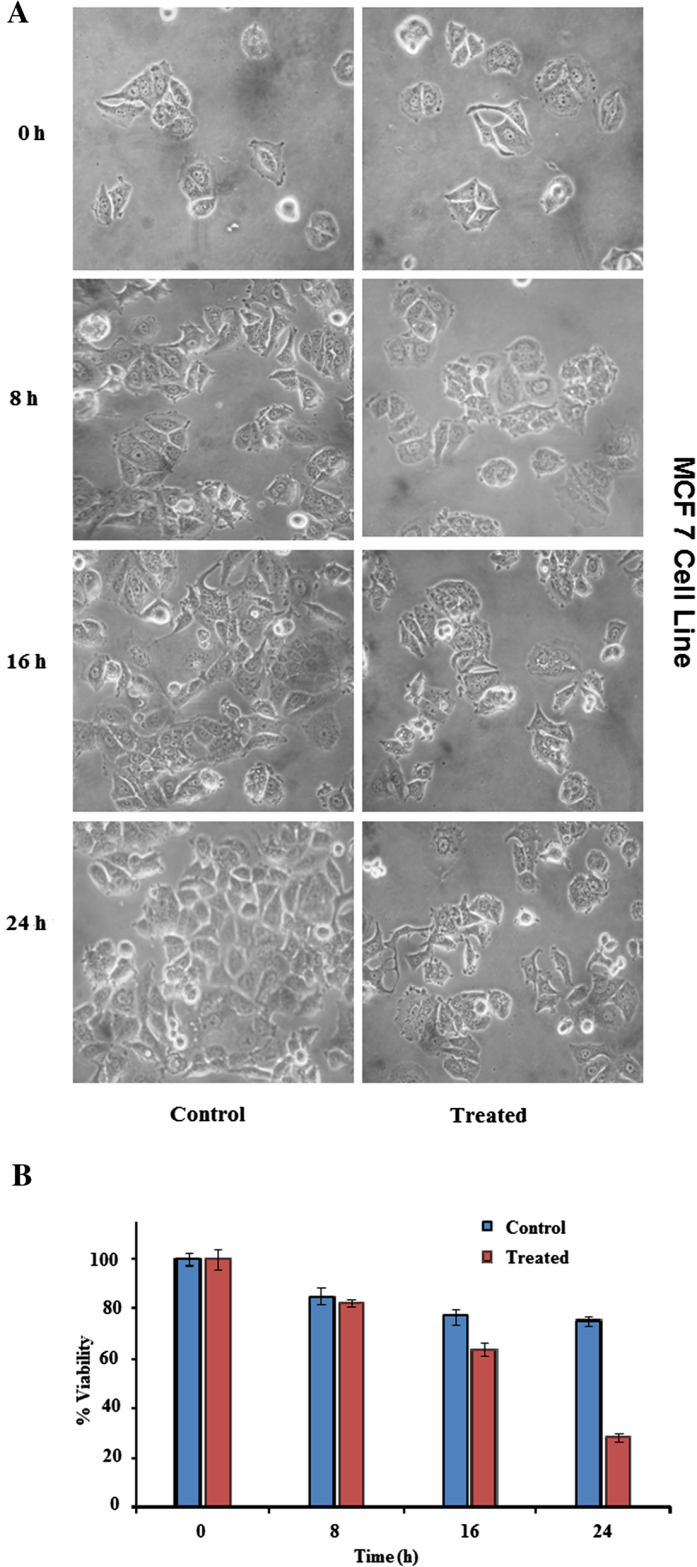
**Time dependent effect of 30 μg/ml of** ***M. macrophylla*** **leaf extract on the proliferation of MCF7, human breast adenocarcinoma, cell line. (A)** observed under phase contrast microscope **(B)** % inhibition in growth was quantified based on Trypan blue dye exclusion assay.

Of the five potential plant extracts, three, *Artemisia indica* (P4), *Maesa macrophylla* (P19) and *Phlogacanthus thyrsiformis* (P22) could inhibit the growth of HeLa and MCF7 cells. The extent of inhibition by P4 and P22 was in the range of 50 to 60 %. On the other hand, P19, showed ~ 75 to 80 % growth inhibition of both the cell lines. The extract of *Eupatorium odoratum* (P16) was cytotoxic to HepG_2_ (53.11 ± 4.87 % inhibition) and HeLa (78.03 ± 1.63) cells, whereas *Eupatorium adenophorum* (P15) could inhibit the growth of only HeLa cells by ~61 % (Table 2). Thus, these five plants were considered to possess potent activity against at least one cancer cell line. The cytotoxicity was also assessed simultaneously at different time points by observing the cells under phase contrast microscope and estimating the % inhibition in growth using Trypan blue dye exclusion assay. The results represented in Figures 1 and 2 clearly show the growth inhibitory effect in both HeLa and MCF7 cell lines in the presence of *Maesa macrophylla* extract. In contrast, a substantial inhibition was not detected when *M. macrophylla* leaf extract was used against HepG_2_ cells (Additional file 1). The results observed with the other four extracts also matched with that obtained using MTT assay (data not shown).

The five promising plant extracts obtained after the first screening were further assessed for their IC_50_ (dose that inhibits cell growth by 50%) values at the concentration range of 50 μg, 40 μg, 30 μg, 20 μg, and 10 μg. To determine the concentration of the plant extracts that caused a 50% reduction in cell viability (IC_50_), regression analysis was done. The results obtained are tabulated in Table 3. The IC_50_ value of *Artemisia indica* was found to be 48 μg/ml for both HeLa and MCF7 cell lines. Earlier studies have demonstrated antimicrobial, cytotoxic and antioxidant activities of essential oil from the aerial parts of *Artemisia indica* [29]. An IC_50_ value of 32 μg/ml for HeLa cells and 50 μg/ml for HepG_2_ was obtained with the extract of *Eupatorium odoratum*. The ability of acetone and ethyl acetate extracts of leaves of *Eupatorium odoratum* to induce cell death in MCF7 and Vero cell lines have previously been reported [30]. In our studies, we did not observe cytotoxicity of *E. odoratum* against MCF7 perhaps because we used ethanol as the extracting solvent. *Phlogacanthus thyrsiformis* is an evergreen shrub (vasaka in hindi) that has been reported to have antimicrobial, analgesic and free radical scavenging properties [31-33]. Our studies revealed cytotoxic activity of *P. thyrsiformis* with IC_50_ value of ~50 μg/ml for two cancer cell lines HeLa and MCF7. *Eupatorium adenophorum* showed an IC_50_ value of 42 μg/ml against HeLa cell lines. The antioxidant activity of essential oil of *E. adenophorum* has earlier been reported [34].

**Table 3:** **Cytotoxic activity expressed as IC**_**50**_ **(μg/ml) of extracts of five selected plants**

**Plant**	**HeLa**	**Hep G2**	**MCF-7**
*Artemisia indica*	48 ± 0.6	>50	48 ± 1.2
*Eupatorium adenophorum*	42 ± 1.49	ND	ND
*Eupatorium odoratum*	32 ± 3.8	50 ± 0.9	ND
*Maesa macrophylla*	22.45 ± 2.5	ND	22.66 ± 1.6
*Phlogacanthus thyrsiformis*	46.7 ± 1.3	ND	49 ± 0.4

Data are presented as mean ± SD from three independent experiments, each run in triplicate. IC_50_ was calculated after exposing the cells to the extracts for 24 h.

ND- Not determined.

**Figure 3:**
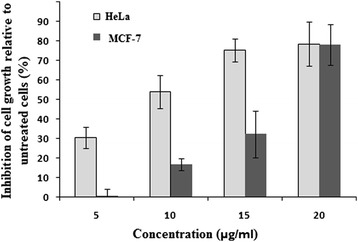
**Dose dependent growth inhibitions of HeLa and MCF7 cell lines after exposing the cells to** ***M. macrophylla*** **leaf extract for 72 h.** Regression analysis was done to calculate IC_50_ values at 72 h.

Among the five plants, *Maesa macrophylla* showed the most promising inhibitory effect at 24 h incubation with IC_50_ value of 22.45 μg/ml and 22.66 μg/ml for HeLa and MCF7 cell lines respectively. *Maesa macrophylla,* belonging to family Myrsinaceae, is found in West Bengal and Uttar Pradesh in India. The bark of the plant is used to treat tonsillitis, the juice of the fruit is applied to treat scabies and the leaves are used to treat fever and boils. The bark of the plant has been reported to possess antimicrobial and antiviral activity [35]. Studies on chemical components of *M. macrophylla* leaves have revealed the presence of a novel quinone [36]. However, detailed analysis of bioactive properties of this plant has not yet been reported. According to the US National Cancer Institute Plant Screening Program, a crude extract is generally considered to have *in vitro* cytotoxic activity against carcinoma cells, if the IC_50_ value at an incubation period between 48 and 72 hours, is less than 20 μg/ml [37]. Based on these criteria, we further evaluated the cytotoxic effects of *Maesa macrophylla* at 72 h of incubation. Our results revealed an IC_50_ of 9.55 μg/ml for HeLa and 16.19 μg/ml for MCF7 cell line (Figure 3). Hence, it follows that, out of the 30 plants screened for their cytotoxicity, only the leaf extract from *Maesa macrophylla* promises to have a high level of cytotoxicity against human breast adenocarcinoma, MCF7 and cervix adenocarcinoma, HeLa cell lines. In a recent report, Fadeyi *et al*. demonstrated that among the twenty four traditionally used Nigerian medicinal plants, one plant exhibited potent cytotoxic activity against five different cancer cell lines including MCF7 (26).

Phytochemicals are considered as active medicinal chemical constituents of plants [38]. To analyse the presence of different constituents in leaves of *M. macrophylla*, the extract was subjected to TLC and detected using specific reagents [39]. The results revealed the presence of various medicinally important phytochemicals such as coumarins, flavonoids, tannins, saponins, terpenes and steroids (Table 4). All these bioactive components have been shown to possess anti cancer properties. For example, Genistein, a coumarin derivative and a natural component of soy, prevents breast and prostate cancers in animal models [40]. Diosgenin, a naturally occurring steroid; fisetin, a flavonoid present in apples and strawberries; and triterpenoids, found in various plants have been shown to inhibit breast cancer [41-43]. The ability of saponins and tannins to exhibit cytotoxic properties also has been reported earlier [44,45]. The isolation of different constituents from *Maesa macrophylla* and evaluating their anticancer properties are underway.

**Table 4:** **Qualitative analysis of phytochemicals in** ***Maesa macrophylla*** **leaf extract**

**Phytochemical**	**Detection system**	**Observation**	**Result**
Coumarin	365 nm	Blue spot	+
Flavonoids	5% AlCl_3_	Yellow spot	+
Tannin	2% FeCl_3_	Greenish Black spot	+
Saponin	10% Vanillin (Ethanol)	Violet and yellow	+
Monoterpene alcohol, Bitter principle saponin	Vanillin-H_2_SO_4_	Blue	+
Triterpene and steroid	Lieberman-Buchard’s reagent	Brown and yellow spots	+

+ tested positive for phytochemicals.

## Conclusions

This is perhaps the first study that deals with the screening of plants from Darjeeling hills for their cytotoxic activity against cancerous cell lines. The outcome of the present study indicates that five out of the thirty plants tested, exerted a >50% growth inhibitory effect on HeLa, MCF7 and HepG_2_ cancer cells. The level of inhibition was different for different cell lines and none of the extract could inhibit all the three cancer cell lines under investigation. Only one plant, *Maesa macrophylla*, showed a highly promising cytotoxic activity against HeLa (IC_50_ 9.55 μg/ml) and MCF7 (IC_50_ 16.19 μg/ml) cell lines. Phytochemical analysis revealed the presence of multiple medicinally active compounds. Identifying the active compound(s) and comparing its cytotoxicity with known anticancer compounds is in progress.

## Additional file

Additional file 1:
**Activity of** ***M. macrophylla*** **leaf extract against HepG**_**2**_ **cells.** HepG_2_ cells were incubated either with DMSO (control) or the extract (treated) and observed under phase contrast microscope at different time points.
