# Supplementary figures and images for: The in vitro cytotoxic activity of ethno-pharmacological important plants of Darjeeling district of West Bengal against different human cancer cell lines
Source: BMC Complement Altern Med. 2015 Feb 7;15:22. doi: 10.1186/s12906-015-0543-5 (PMC4331178; doi:10.1186/s12906-015-0543-5)

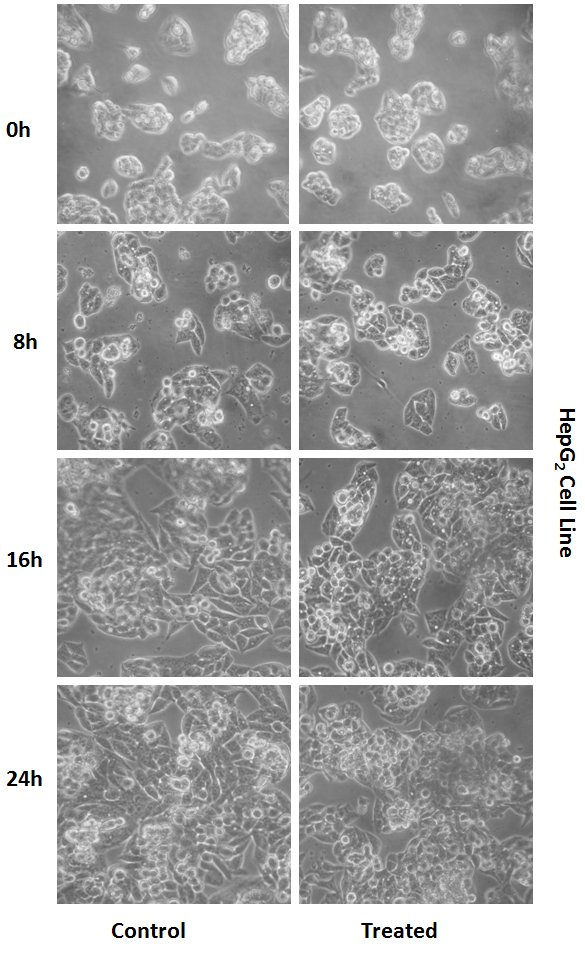

Supplement: Additional file 1: — Activity of M. macrophylla leaf extract against HepG 2 cells. HepG2 cells were incubated either with DMSO (control) or the extract (treated) and observed under phase contrast microscope at different time points. [file 12906_2015_543_MOESM1_ESM.tiff]
